# Plasma Dickkopf-1 Levels Are Associated with Chronic Kidney Disease

**DOI:** 10.3390/metabo15050300

**Published:** 2025-04-30

**Authors:** Yu-Hsuan Li, Yu-Cheng Cheng, Junyi Wu, I-Te Lee

**Affiliations:** 1 Department of Digital Medicine, Taichung Veterans General Hospital, Taichung 407219, Taiwan; brightlight@vghtc.gov.tw; 2 Department of Computer Science & Information Engineering, National Taiwan University, Taipei 106319, Taiwan; 3 School of Medicine, National Yang Ming Chiao Tung University, Taipei 112304, Taiwan; fererro1552@vghtc.gov.tw; 4 Institute of Biomedical Sciences, National Chung Hsing University, Taichung 402002, Taiwan; 5 Division of Endocrinology and Metabolism, Department of Internal Medicine, Taichung Veterans General Hospital, Taichung 407219, Taiwan; junyiwu@vghtc.gov.tw; 6 School of Medicine, Chung Shan Medical University, Taichung 402306, Taiwan; 7 Department of Post-Baccalaureate Medicine, College of Medicine, National Chung Hsing University, Taichung 402002, Taiwan

**Keywords:** chronic kidney disease, Dickkopf-1, fibrosis, Wnt

## Abstract

Background: Wnt/β-catenin signaling is important in the development and repair of the kidney. Dickkopf-1 (DKK-1) is characterized as an inhibitor of the Wnt/β-catenin signaling pathway. Purpose: We examined the relationship between plasma DKK-1 levels and the risk of chronic kidney disease (CKD). Methods: In this cross-sectional study, patients without known diabetes mellitus who were admitted for coronary angiography due to angina were enrolled. Fasting blood samples were collected at a predetermined outpatient visit. Results: Among 373 enrolled patients, 62 (16.6%) were in the CKD group, and 311 (83.4%) were in the nonCKD group. Plasma DKK-1 levels were significantly higher in the CKD group than in the nonCKD group (697.2 ± 174.7 vs. 589.0 ± 193.3 pg/mL; *p* < 0.001). Plasma DKK-1 levels were inversely correlated with the eGFR (Pearson’s correlation coefficient = −0.265; *p* < 0.001). On the basis of multivariable logistic regression analyses, patients in the highest DKK-1 quartile had a significantly greater risk of CKD (OR = 4.188; 95% CI: 1.564, 11.212; *p* = 0.004) than did those in the lowest DKK-1 quartile. Conclusions: Plasma DKK-1 levels are associated with the risk of CKD in patients with angina. Further studies investigating the underlying mechanisms involved in the relationship between DKK-1 and CKD are warranted.

## 1. Introduction

Chronic kidney disease (CKD) is prevalent worldwide, and approximately 674 million patients with CKD were reported globally in 2021 [1]. CKD has been a heavy burden on global health in the past 30 years, with an increasing trend of mortality and disability resulting from CKD [1,2]. Although diabetes mellitus (DM) is an important cause of CKD, most (more than 70%) CKD cases are due to unspecified causes [1].

Wnt/β-catenin signaling plays a crucial role in kidney development [3]. Moreover, Wnt/β-catenin signaling may play a role in maintaining renal function and regeneration after acute kidney injury in adults [4]. However, dysregulation of Wnt/β-catenin signaling can induce renal fibrosis and CKD progression [5]. Moreover, the Wnt/β-catenin signaling pathway is reportedly associated with cardiorenal syndrome [6,7].

Dickkopf-1 (DKK-1), a secreted glycoprotein, is characterized as an inhibitor of the canonical β-catenin-dependent Wnt pathway [8]. Recently, we reported that plasma DKK-1 levels are predictive of major adverse cardiac events (MACEs) in patients with angina [9]. Downregulation of DKK-1 expression was shown to decrease renal fibrosis in a streptozotocin-induced diabetic model of rats [10]. In contrast, inhibition of Wnt/β-catenin signaling via delivery of the DKK-1 gene can decrease fibrosis after obstructive injury to the kidney in mice [11]. Therefore, we aimed to examine the relationship between circulating DKK-1 levels and the risk of CKD in a cross-sectional study.

## 2. Materials and Methods

### 2.1. Study Design and Population

In this cross-sectional study, we enrolled adults who were admitted for selective coronary angiography due to angina. Subjects were excluded from enrollment if they had been diagnosed with DM; had severe systemic diseases, including infection or inflammation; or were pregnant. This study was approved by the Institutional Review Board of Taichung Veterans General Hospital and complied with the Declaration of Helsinki. After the participants provided written informed consent, an outpatient interview was arranged for the study procedure. Furthermore, subjects were excluded from the analyses if they had previously undergone coronary intervention treatment before admission. After the anthropometric measurements, blood and urine samples were collected in the morning after overnight fasting.

### 2.2. Measurement

Plasma DKK-1 levels were measured using an immunoassay kit (R&D Systems, Minneapolis, MN, USA) with an interassay coefficient of variation (CV) of 8.1% and an intraassay CV of 2.6%. Plasma glucose was measured by the oxidative peroxidase method (Wako Diagnostics, Tokyo, Japan). HbA1c levels were measured using boronate affinity high-performance liquid chromatography (NGSP certified, Primus Corp., Kansas City, MO, USA). Serum levels of creatinine, high-sensitivity C-reactive protein (hsCRP), and lipids were measured using commercial kits (Beckman Coulter, Fullerton, CA, USA). The estimated glomerular filtration rate (eGFR) was calculated using the Chronic Kidney Disease Epidemiology Collaboration equation [12]. CKD was defined as an eGFR < 60 mL/min/1.73 m^2^ [13]. The urine albumin-to-creatinine ratio (UACR) was calculated by dividing the levels of urine albumin (mg) by those of urine creatinine (g), and increased albuminuria was defined as a UACR ≥ 30 mg/g. Obesity was defined as BMI ≥ 27 kg/m^2^ [14]. Central obesity was defined as a waist circumference > 90 cm in men or >80 cm in women [15]. Metabolic syndrome was defined on the basis of the American Heart Association and National Heart, Lung, and Blood Institute Scientific Statement [16]. Hypertension was defined as a systolic blood pressure ≥ 130 mmHg, a diastolic blood pressure ≥ 80 mmHg, or a history of antihypertensive medication use. CAD was defined as a history of MI and/or a coronary lesion with lumen narrowing ≥ 50% according to angiography during this hospitalization.

### 2.3. Statistical Analysis

Continuous variables are presented as the means ± standard deviations, and categorical variables are presented as numbers (percentages). We examined the statistical significance of the between-group differences using independent t tests for continuous variables and chi-square tests for categorical variables. The correlation coefficient between DKK-1 and the eGFR was determined using Spearman’s rank correlation. Receiver operating characteristic (ROC) curve analysis was performed to differentiate CKD according to the DKK-1 levels.

We further divided all of the enrolled participants into four quartiles on the basis of their DKK-1 levels to investigate the trend of CKD prevalence across different DKK-1 levels. Trend analysis was used to evaluate differences across the DKK-1 quartiles. Logistic regression analyses were performed to estimate the odds ratios (ORs) and the associated 95% confidence intervals (CIs) for the risk of CKD of the higher quartiles of DKK-1 compared with the lowest quartile of DKK-1 after adjusting for potential confounding variables that were significantly associated with both CKD status and DKK-1 levels. A two-sided *p* value < 0.05 was considered to indicate statistical significance. Statistical analysis was conducted using SPSS v22.0 (IBM, Armonk, NY, USA), and the area under the curve in the multivariable model was calculated using Python 3.10.

## 3. Results

In the present study, a total of 373 participants were enrolled, including 149 subjects with an eGFR ≥ 90 mL/min/1.73 m^2^, 162 subjects with an eGFR between 60 and 89.9 mL/min/1.73 m^2^, 57 subjects with an eGFR between 30 and 59.9 mL/min/1.73 m^2^, 2 subjects with an eGFR between 15.0 and 29.9 mL/min/1.73 m^2^, and 3 subjects with an eGFR < 15 mL/min/1.73 m^2^. No subjects receiving renal replacement therapy were enrolled. Therefore, 62 (16.6%) subjects were categorized into the CKD group and 311 (83.4%) into the nonCKD group. Table 1 shows the baseline characteristics of the participants in the CKD and nonCKD groups. The subjects were significantly older in the CKD group than in the nonCKD group (68.5 ± 10.7 vs. 58.2 ± 10.8 years; *p* < 0.001). The subjects in the CKD group had significantly higher systolic blood pressure than those in the nonCKD group (132.7 ± 17.5 vs. 127.0 ± 17.5 mmHg; *p* = 0.020). The hsCRP levels were significantly greater in the CKD group than in the nonCKD group (3.5 ± 3.2 vs. 2.1 ± 2.2 mg/L; *p* < 0.001). The prevalence of an increased UACR was significantly greater in the CKD group than in the nonCKD group (35.5% vs. 10.9%; *p* < 0.001). The proportion of subjects using diuretics was significantly greater in the CKD group than in the nonCKD group (25.8% vs. 11.6%; *p* = 0.006). The plasma levels of DKK-1 were significantly greater in the CKD group than in the nonCKD group (697.2 ± 174.7 vs. 589.0 ± 193.3 pg/mL; *p* < 0.001).

**Figure 1:**
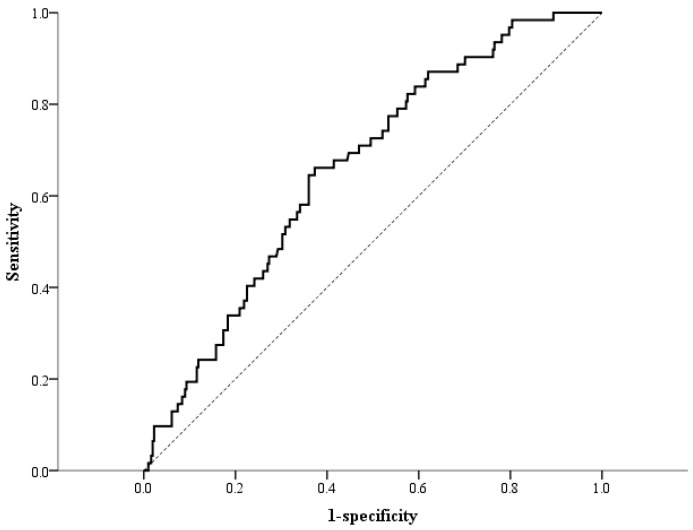
Receiver operating characteristic (ROC) curves for differentiating chronic kidney disease (CKD) status on the basis of plasma Dickkopf-1 levels. The area under the curve was 0.661 (95% CI: 0.593; 0.729; *p* < 0.001). The dashed diagonal line represents the line of no discrimination.

**Figure 2:**
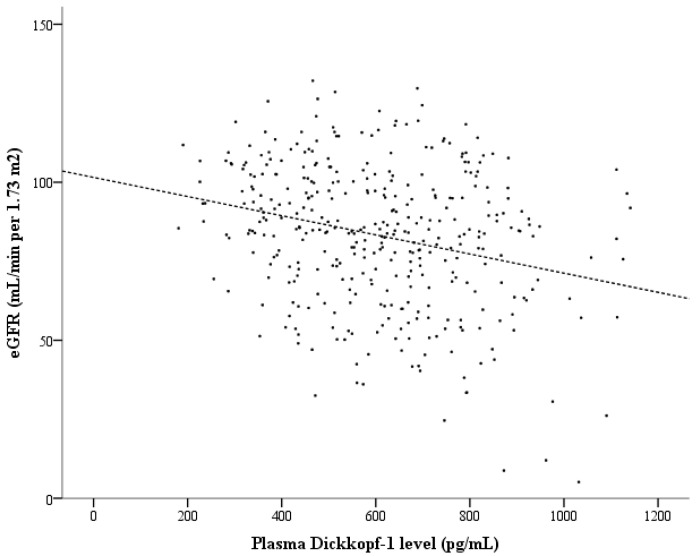
The significant inverse correlation between the estimated glomerular filtration rate (eGFR) and plasma Dickkopf-1 levels. The Spearman’s rank correlation coefficient was −0.265 (*p* < 0.001).

**Figure 3:**
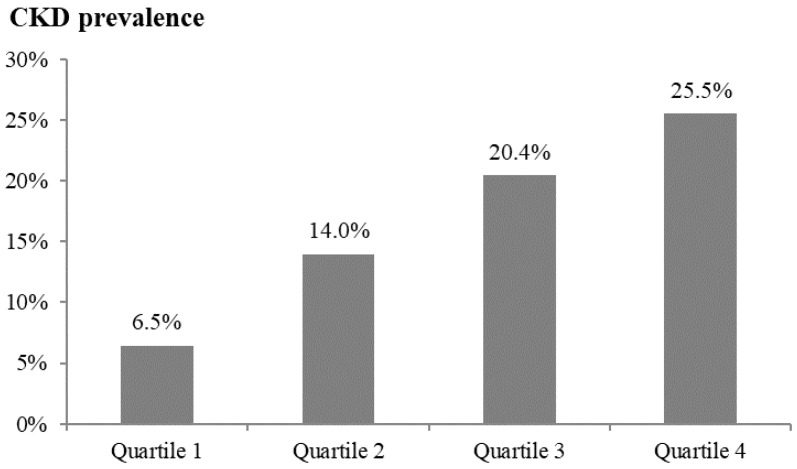
The prevalence of chronic kidney disease (CKD) presented as quartiles of plasma Dickkopf-1 levels (*p* value for trend < 0.001). The ranges of Dickkopf-1 are as follows: quartile 1 (*n* = 93) between 180.7 and 456.7; quartile 2 (*n* = 93) between 456.8 and 608.5; quartile 3 (*n* = 93) between 608.6 and 747.5; and quartile 4 (*n* = 94) between 747.6 and 1141.1.

On the basis of the ROC curve for differentiating CKD (Figure 1), a cutoff value of 641.8 pg/mL for plasma DKK-1 levels provided a sensitivity of 66.1% and specificity of 66.7% for differentiating CKD (area under the curve = 0.661; 95% CI: 0.593–0.729; *p* < 0.001). According to Spearman’s rank correlation, plasma DKK-1 levels were inversely correlated with the eGFR (correlation coefficient [σ] = −0.265; *p* < 0.001; Figure 2). In addition, plasma DKK-1 levels were also significantly correlated with age (σ = 0.142; *p* = 0.006), total cholesterol (σ = 0.112; *p* = 0.031), triglycerides (σ = 0.192; *p* < 0.001), and UACR (σ = 0.113; *p* = 0.029; Table 2). To examine the increasing trend of plasma DKK-1 levels toward CKD, we further divided all of the subjects into four groups on the basis of plasma DKK-1 quartiles. The prevalence of CKD showed a significant increase from the lowest to the highest DKK-1 quartiles (*p* value for trend < 0.001, Figure 3).

To assess the factors associated with plasma DKK-1 levels, we compared DKK-1 levels between the dichotomous groups of CKD risk factors (Table 3). Higher plasma DKK-1 levels were observed in patients with lower HDL cholesterol and higher triglyceride and hsCRP levels (*p* = 0.009, 0.032, and 0.007, respectively). Notably, hsCRP should be considered a confounding factor because of its significant associations with CKD status (Table 1) and DKK-1 levels (Table 3). After adjustment for age, sex, and hsCRP levels, CKD risk was significantly associated with the DKK-1 quartile (*p* = 0.024). Furthermore, the subjects in the fourth (highest) DKK-1 quartile group had the highest risk of CKD (OR = 4.188; 95% CI: 1.564, 11.212; *p* = 0.004), followed by those in the third quartile (OR = 3.580, 95% CI: 1.307; 9.801; *p* = 0.013), and then by those in the first (lowest) DKK-1 quartile group (Table 4). However, there was no significant difference in CKD risk between the second and lowest quartiles of DKK-1 (*p* > 0.05). On the basis of the ROC curve, the area under the curve for differentiating CKD is significantly increased by adding DKK-1 data with a cutoff of 641.8 pg/mL in the age + sex + hsCRP model (0.735 vs. 0.697; difference = 0.038; 95% CI = 0.008–0.066; *p* = 0.007).

## 4. Discussion

Our main finding in the present study is that plasma DKK-1 levels are inversely correlated with the eGFR in patients who have undergone coronary angiography for angina. Moreover, high plasma DKK-1 levels are significantly associated with CKD risk. Similarly, Wang et al. [17] reported that the mean plasma DKK-1 levels were significantly greater in 50 patients with lupus nephritis than in 40 healthy controls. DKK-1 can increase the expression of profibrotic factors in mesangial cells in a hyperglycemic model and induce renal fibrosis in streptozotocin-induced diabetic rats [18]. Therefore, increased plasma DKK-1 levels may reflect renal fibrosis induced by dysregulation of the Wnt/β-catenin signaling pathway.

Mihai et al. [19] reported that 24 biomarkers were significantly associated with CKD status on the basis of 105 proteins assessed using the Proteome Profiler Cytokine Array Kit in 76 subjects, and DKK-1 was included among the CKD-associated biomarkers. The relationship between DKK-1 levels and CKD status might involve inflammatory or mineral biomarkers. However, in contrast to our results, serum DKK-1 levels were significantly lower in patients with CKD than in controls [19]. Behets et al. [20] reported that serum DKK-1 levels were significantly lower in patients with CKD than in controls without CKD, but serum DKK-1 levels were not significantly different across different CKD stages in patients not on dialysis. Hamada-Ode et al. [21] reported that serum DKK-1 levels were significantly lower in Japanese individuals with an eGFR < 30 mL/min/1.73 m^2^ than in those with an eGFR ≥ 30 mL/min/1.73 m^2^. Discrepantly, Hsu et al. [22] reported that serum DKK-1 levels were significantly greater in patients with an eGFR < 30 mL/min/1.73 m^2^ than in healthy controls, and that serum DKK-1 levels were predictive of the onset of end-stage renal disease in patients with CKD during an eight-year follow-up. Serum DKK-1 levels vary widely across studies, and the variation in serum DKK-1 levels might result from differences in blood platelet counts [20].

In the present study, plasma DKK-1 levels were not significantly different between patients with and without obstructive coronary artery disease. Similarly, Wang et al. [23] reported that plasma DKK-1 levels were not significantly associated with vessel number or the stenosis degree of coronary arteries in patients with acute coronary syndrome. However, plasma DKK-1 was a significant predictor of MACE during a median two-year follow-up. Since CKD is a prognostic factor in patients with CAD, the relationship between DKK-1 levels and CKD may play a role in the prognosis of CAD. Furthermore, the mean plasma DKK-1 level of patients without CKD in the present study is similar to those reported in other studies, e.g., those reported in healthy controls and subjects who did not experience MACE [17,23]. Therefore, the plasma level, rather than the serum level, of DKK-1 could be a biomarker for CKD.

DKK-1 plays an important role in chronic inflammation [24,25]. A high CRP level has been thought to indicate a proinflammatory state and to be a risk factor of cardiovascular disease [26]. In the present study, plasma DKK-1 was significantly associated with hsCRP levels. In line with our findings, Wang et al. [23] reported that plasma DKK-1 levels were positively correlated with hsCRP levels in patients with acute coronary syndrome. Because hsCRP can facilitate the epithelial–mesenchymal transition and promote fibrosis through the Wnt/β-catenin signaling pathway in proximal tubular cells, decreases in mesangial matrix deposition and glomerular basement membrane thickness were observed in streptozotocin-induced diabetic rats after the knockdown of hsCRP [27]. Our results showed that plasma DKK-1 levels are an independent factor for CKD after adjusting for hsCRP levels.

In the present study, dyslipidemia status, characterized by hypertriglyceridemia and low HDL cholesterol levels, was associated with plasma DKK-1 levels. Goliasch et al. [28] reported that high Wnt-1 protein levels are associated with dyslipidemia in patients after myocardial infarction. Moreover, Wnt activation has been reported to inhibit adipocyte formation, and DKK-1 overexpression can attenuate the effects of Wnt and promote lipogenesis in mice with obesity induced by a high-fat diet [29]. Furthermore, metabolic syndrome, characterized by central obesity, high blood pressure, high fasting glucose, and dyslipidemia, is associated with meta-inflammation and chronic kidney disease [30]. However, dyslipidemia status was not significantly associated with CKD risk in the present study.

The strengths of our study include demonstrating that plasma DKK-1 levels are inversely correlated with the eGFR and that the Wnt/β-catenin signaling pathway may be involved in the underlying mechanism of CKD in patients without known DM. However, the area under the ROC curve for differentiating CKD was only 0.661 according to the plasma DKK-1 levels in the present study. The etiology of CKD is complex, and new biomarkers may provide further information to enable us to better understand the underlying mechanisms. There are several limitations in the present study. First, we did not investigate the real mechanism underlying the relationship between DKK-1 levels and CKD. Second, we did not examine the source of increased DKK-1 protein in the plasma. Third, we did not investigate whether reducing DKK-1 as a treatment target can prevent CKD development. Fourth, we did not identify the real etiology of CKD, which should be proven by biopsy. Finally, we enrolled patients with angina, a population at high risk of developing CKD, so our findings cannot be expanded to other populations.

## 5. Conclusions

Plasma DKK-1 levels are associated with CKD in patients with angina. Because DKK-1 is a potential antagonist of the Wnt/β-catenin signaling pathway, further studies to investigate the role of dysregulated Wnt/β-catenin signaling in the development of CKD are warranted.

## Figures and Tables

**Table 1:** Characteristics of enrolled patients grouped by CKD status.

	CKD (*n* = 62)		nonCKD (*n* = 311)		*p*
Age (year)	68.5	±10.7	58.2	±10.8	<0.001
Male, *n* (%)	49	(79.0%)	238	(76.5%)	0.793
Current smoker, *n* (%)	17	(27.4%)	106	(34.1%)	0.384
CAD, *n* (%)	37	(59.7%)	151	(48.6%)	0.144
Waist circumference (cm)	90.2	±8.7	91.0	±9.7	0.578
BMI (kg/m^2^)	25.4	±3.2	26.3	±3.9	0.078
Systolic BP (mmHg)	132.7	±17.5	127.0	±17.5	0.020
Diastolic BP (mmHg)	73.2	±10.7	74.9	±10.3	0.257
DKK-1 (pg/mL)	697.2	±174.7	589.0	±193.3	<0.001
Fasting glucose (mmol/L)	5.3	±1.1	5.3	±0.7	0.847
HbA1c (%)	5.8	±0.6	5.8	±0.6	0.828
Total cholesterol (mmol/L)	4.5	±0.9	4.5	±1.0	0.795
HDL cholesterol (mmol/L)	1.2	±0.3	1.3	±0.3	0.558
Triglycerides (mmol/L)	1.6	±1.2	1.5	±0.9	0.514
eGFR (mL/min/1.73 m^2^)	53.7	±14.1	90.3	±23.5	<0.001
C-reactive protein (mg/L)	3.5	±3.2	2.1	±2.2	<0.001
Increased UACR, *n* (%)	22	(35.5%)	34	(10.9%)	<0.001
Metabolic syndrome, *n* (%)	24	(38.7%)	138	(44.4%)	0.496
Use of antiplatelet agents, *n* (%)	56	(90.3%)	295	(94.9%)	0.231
Use of statins, *n* (%)	31	(50.0%)	148	(47.6%)	0.835
Hypertension, *n* (%)	60	(96.8%)	289	(92.9%)	0.396
Use of antihypertensive agents, *n* (%)	53	(85.5%)	269	(86.5%)	0.993
ACE inhibitor or ARB, *n* (%)	38	(61.3%)	159	(51.1%)	0.185
α-blocker, *n* (%)	5	(8.1%)	11	(3.5%)	0.159
β-blocker, *n* (%)	19	(30.6%)	83	(26.7%)	0.630
Calcium channel blocker, *n* (%)	35	(56.5%)	159	(51.1%)	0.530
Diuretics, *n* (%)	16	(25.8%)	36	(11.6%)	0.006

ACE, angiotensin-converting enzyme; ARB, angiotensin II receptor blocker; BMI, body mass index; BP, blood pressure; CAD, coronary artery disease; CKD, chronic kidney disease; DKK-1, Dickkopf-1; eGFR, estimated glomerular filtration rate; HbA1c, glycated hemoglobin; HDL, high-density lipoprotein; UACR, urinary albumin-to-creatinine ratio.

**Table 2:** Spearman’s rank correlation coefficients (σ) between plasma DKK-1 and continuous variables of CKD risk factors.

Continuous Variable	σ	*p*
Age	0.142	0.006
Waist	−0.014	0.787
BMI	−0.073	0.157
Systolic BP	0.054	0.298
Diastolic BP	0.005	0.920
Fasting glucose	−0.005	0.931
HbA1c	0.069	0.184
Total cholesterol	0.112	0.031
HDL cholesterol	−0.095	0.066
Triglycerides	0.192	<0.001
C-reactive protein	0.094	0.068
UACR	0.113	0.029

BMI, body mass index; BP, blood pressure; CKD, chronic kidney disease; DKK-1, Dickkopf-1; eGFR, estimated glomerular filtration rate; HbA1c, glycated hemoglobin; HDL, high-density lipoprotein; UACR, urinary albumin-to-creatinine ratio.

**Table 3:** Plasma DKK-1 levels in patients grouped according to CKD risk factors.

Variable	Group	Patient Number	Mean	±SD	Difference in Mean (95% CI)			*p*
Age	<60 years	182	589.3	±199.7	−34.6	(−74.1, 4.9)		0.086
	≥60 years	191	623.9	±188.1				
Sex	Female	86	611.1	±184.2	5.3	(−41.8, 52.3)		0.825
	Male	287	605.8	±197.6				
Current smoker	No	250	593.2	±196.6	−41.8	(−83.7, 0.1)		0.051
	Yes	123	635.0	±187.5				
CAD	No	185	612.0	±191.2	9.9	(−29.8, 49.5)		0.625
	Yes	188	602.1	±197.8				
Hypertension	No	24	611.3	±229.4	4.6	(−76.2, 85.3)		0.911
	Yes	349	606.7	±192.1				
Central obesity *	No	161	608.6	±188.6	2.8	(−37.2, 42.8)		0.890
	Yes	212	605.8	±199.0				
BMI	<27 kg/m^2^	248	614.3	±191.0	21.6	(−20.3, 63.6)		0.310
	≥27 kg/m^2^	125	592.6	±200.9				
Systolic BP	<130 mmHg	202	595.7	±197.8	−24.7	(−64.4, 14.9)		0.221
	≥130 mmHg	171	620.4	±189.9				
Diastolic BP	<80 mmHg	258	612.5	±201.1	17.8	(−25.1, 60.6)		0.416
	≥80 mmHg	115	594.7	±178.5				
Fasting glucose	<7.2 mmol/L	52	574.8	±186.1	−31.6	(−90.8, 27.7)		0.296
	≥7.2 mmol/L	255	606.3	±200.3				
HbA1c	<8.5%	274	605.9	±194.9	−4.0	(−48.8, 40.9)		0.862
	≥8.5%	99	609.9	±193.7				
Total cholesterol	<4.14 mmol/L	139	598.9	±212.0	−13.0	(−53.9, 28.0)		0.533
	≥4.14 mmol/L	234	611.8	±183.4				
Low HDL cholesterol ^#^	No	269	590.8	±188.8	−58.2	(−102.0, −14.5)		0.009
	Yes	104	649.0	±203.0				
Triglycerides	<1.7 mmol/L	253	592.1	±191.2	−46.2	(−88.4, −4.1)		0.032
	≥1.7 mmol/L	120	638.3	±198.1				
C-reactive protein	<2 mg/L	216	584.0	±181.9	−54.7	(−94.4, −15.0)		0.007
	≥2 mg/L	157	638.7	±206.7				
UACR	<30 mg/g	317	601.3	±193.1	−38.1	(−93.4, 17.3)		0.177
	≥30 mg/g	56	639.3	±200.0				
Metabolic syndrome	No	211	592.5	±191.6	−33.5	(−73.3, 6.3)		0.099
	Yes	162	625.9	±196.9				
Use of statins	No	194	607.9	±187.0	1.9	(−37.7, 41.6)		0.923
	Yes	179	606.0	±202.5				
Use of antihypertensive drugs	No	51	628.8	±214.9	25.3	(−32.4, 82.9)		0.389
	Yes	322	603.5	±191.0				
Use of antiplatelet drugs	No	22	584.9	±222.4	−23.5	(−107.6, 60.6)		0.583
	Yes	351	608.4	±192.7				
ACE inhibitor or ARB	No	176	617.6	±190.6	20.2	(−19.5, 59.8)		0.318
	Yes	197	597.5	±197.7				
α-blocker	No	357	608.5	±195.1	34.0	(−63.8, 131.7)		0.495
	Yes	16	574.5	±178.7				
β-blocker	No	271	603.7	±195.9	−12.1	(−56.5, 32.4)		0.594
	Yes	102	615.8	±190.9				
Calcium channel blocker	No	179	601.7	±194.0	−10.2	(−49.8, 29.5)		0.614
	Yes	194	611.9	±195.0				
Diuretics	No	321	606.3	±194.8	−5.0	(−62.2, 52.2)		0.864
	Yes	52	611.3	±193.4				

ACE, angiotensin-converting enzyme; ARB, angiotensin II receptor blocker; BMI, body mass index; BP, blood pressure; CAD, coronary artery disease; CKD, chronic kidney disease; DKK-1, Dickkopf-1; eGFR, estimated glomerular filtration rate; HbA1c, glycated hemoglobin; HDL, high-density lipoprotein; SD, standard deviation; UACR, urinary albumin-to-creatinine ratio. * Central obesity means waist circumference >90 cm in men or >80 cm in women. ^#^ Low HDL cholesterol means <40 mg/dL (1.0 mmol/L) in men or <50 mg/dL (1.3 mmol/L) in women.

**Table 4:** Odds ratios (95% CI) for chronic kidney disease (CKD) by quartiles of Dickkopf-1 levels.

	Quartile 1 *n* = 93 (180.7–456.7 pg/mL)	Quartile 2 *n* = 93 (456.8–608.5 pg/mL)	Quartile 3 *n* = 93 (608.6–747.5 pg/mL)	Quartile 4 *n* = 94 (747.6–1141.1 pg/mL)	*p*
CKD/nonCKD	6/87	13/80	19/74	24/70	
Crude	1.000 (reference)	2.356 (0.855, 6.494) **	3.723 (1.413, 9.809) **	4.971 (1.926, 12.833) **	0.006
Model 1	1.000 (reference)	2.173 (0.765, 6.173)	3.769 (1.381, 10.285) **	4.437 (1.664, 11.829) **	0.014
Model 2	1.000 (reference)	2.193 (0.771, 6.235)	3.580 (1.307, 9.801) *	4.188 (1.564, 11.212) **	0.024

Model 1: adjusted for age and sex. Model 2: adjusted for age, sex, and C-reactive protein. * *p* < 0.05; ** *p* < 0.01.

## Data Availability

The datasets used and/or analyzed during the current study are available from the corresponding author on reasonable request.

## Abbreviations

CI	Confidence interval
CKD	Chronic kidney disease
hsCRP	High-sensitivity C-reactive protein
CV	Coefficient of variation
DKK-1	Dickkopf-1
DM	Diabetes mellitus
eGFR	Estimated glomerular filtration rate
MACE	Major adverse cardiac event
OR	Odds ratio
ROC	Receiver operating characteristic
UACR	Urine albumin-to-creatinine ratio
CI	Confidence interval
